# Shengma Biejia Decoction Inhibits Cell Growth in Multiple Myeloma by Inducing Autophagy-Mediated Apoptosis Through the ERK/mTOR Pathway

**DOI:** 10.3389/fphar.2021.585286

**Published:** 2021-03-29

**Authors:** Huibo Dai, Bangyun Ma, Xingbin Dai, Jie Pang, Jingyu Wang, Yandong Zhao, Mengya Wang, Hong Zhang, Haoran Gao, Shushu Qian, Fang Tian, Xuemei Sun

**Affiliations:** ^1^ Affiliated Hospital of Nanjing University of Chinese Medicine, Nanjing, China; ^2^ Department of Hematology, Affiliated Hospital of Nanjing University of Chinese Medicine, Nanjing, China; ^3^ Research Center of Chinese Medicine, Affiliated Hospital of Nanjing University of Chinese Medicine, Nanjing, China

**Keywords:** shengma biejia decoction, multiple myeloma, autophagy, apoptosis, ERK/mTOR pathway

## Abstract

Shengma Biejia decoction (SMBJD), a traditional Chinese formula recorded in the *Golden Chamber*, has been widely used for the treatment of malignant tumors. However, its underlying molecular targets and mechanisms are still unclear. This study showed that SMBJD inhibited tumor growth and stimulated hemogram recovery significantly in a multiple myeloma xenograft model. Western blot and immunohistochemistry assays of tumor tissues showed that SMBJD reduced the ratio of autophagy-related proteins LC3-II/LC3-I, while P62 and apoptosis-related proteins cleaved caspase-3/caspase-3 and Bax/Bcl-2 were upregulated. *In vitro* experiments demonstrated the time-dependent and dose-dependent cytotoxicity of SMBJD on multiple myeloma cell lines H929 and U266 through MTT assays. The LC3-II/LC3-I ratio and number of GFP-LC3 puncta showed that SMBJD inhibited the autophagy process of H929 and U266 cells. Moreover, both SMBJD and 3-methyladenine (3-MA) caused a decrease in LC3-II/LC3-I, and SMBJD could not reverse the upregulation of LC3-II/LC3-I caused by bafilomycin A1 (Baf-A1). Furthermore, the results of annexin V-FITC and propidium iodide double staining demonstrated that SMBJD treatment induced the apoptosis of H929 and U266 cells. These data prove that SMBJD inhibits autophagy and promotes apoptosis in H929 and U266 cells. The results also show that rapamycin could reduce the rate of SMBJD-induced apoptosis in H929 and U266 cells, at a concentration which had no effect on apoptosis but activated autophagy. In addition, analysis of the mechanism indicated that levels of phosphorylated ERK and phosphorylated mTOR were increased by treatment with SMBJD *in vivo* and *in vitro*. These results indicate that SMBJD, an old and effective herbal compound, could inhibit the viability of H929 and U266 cells and induce autophagy-mediated apoptosis through the ERK/mTOR pathway. Thus, it represents a potential therapy strategy for multiple myeloma.

## Introduction

Multiple myeloma (MM) is the second most common hematological malignancy, with an incidence that has exceeded that of acute leukemia in many countries (Kazandjian, 2016; Liu et al., 2019). Over the past few decades, despite the development of several drugs and an in-depth study of its pathogenesis, MM is still considered to be incurable, with almost all patients experiencing recurrence and drug resistance (Laubach et al., 2011; Kohler et al., 2018; Pinto et al., 2020). In addition, the side effects of traditional treatments greatly lower patients’ quality of life (Morawska et al., 2015; McCullough et al., 2018). Thus, developing new treatments for MM is imperative.

Traditional Chinese medicine (TCM) is treasured by the Chinese nation and has been used as a clinical adjuvant treatment for several malignant tumors. It has been demonstrated that TCM can extenuate chemotherapy-related side effects and enhance the efficacy of chemotherapy drugs. For instance, BuPiHeWei decoction, a classic TCM prescription, exerted a protective role in 5-Fu-induced intestinal mucosal injury in rats by regulating the mechanisms of the TLR-4/NF-κB signaling pathway (Sun et al., 2019). Huangqin Gegen decoction, another classical TCM compound, enhanced 5-fluorouracil anti-tumor activity through modulation of the E2F1/TS pathway (Liu et al., 2018). Shengma Biejia decoction (SMBJD) comes from the *Golden Chamber* (also called JinGuiYaoLue) written by Zhongjing Zhang. It is a classic prescription which has been used for the treatment of dermatosis, immunological diseases, and neoplastic disease. Preliminary clinical and basic research has demonstrated that SMBJD combined with the CAG regimen (cytarabine, aclarubicin, and G-CSF) in the treatment of elderly patients with acute myeloid leukemia can improve overall clinical efficacy and reduce the side effects of chemotherapy (Dai et al., 2016). Subsequently, a series of basic studies confirmed that SMBJD could inhibit the proliferation of human acute myeloid leukemia HL-60 cells and induce apoptosis by regulating the MAPK signaling pathway. Moreover, non-cytotoxic concentrations (2, 4, and 8 mg/ml) of SMBJD could decrease cellular migration, chemotaxis, and tube formation of human umbilical vein endothelial cells in a time and dose-dependent manner (Wang et al., 2020). In addition, SMBJD has been used as an adjuvant treatment for MM, with remarkable clinical effects. However, TCM compounds often have multiple ingredients with multiple targets. Thus, the underlying molecular mechanism of SMBJD in MM need to be elucidated.

Autophagy is a physiological process by which cells degrade cellular abnormal proteins and organelles through strict regulatory mechanisms (Wang et al., 2015). A certain level of autophagy is conducive to cell survival (Anding and Baehrecke, 2015). However, it is a double-edged sword for tumor cells (Shintani and Klionsky, 2004). In the early stages of tumorigenesis, the body suppresses the occurrence of precancerous lesions through actuating autophagic cell death of cancer cells (Cai et al., 2018; Wang et al., 2019b) Nevertheless, once the tumor begins to develop, cancer cells begin to resist intracellular and environmental stresses, leading to unlimited proliferation and migration (Mathew et al., 2007; Kimmelman and White, 2017). Furthermore, autophagy can protect tumor cells from chemotherapy drugs, resulting in multi-drug resistance (Buchser et al., 2012; Kumar et al., 2012). MM cells have been shown to have higher autophagy levels than normal plasma cells, which they rely on to handle the overproduction of immunoglobulins (Yang et al., 2012; Yun et al., 2017). Therefore, exploring the treatment of MM through the targeted regulation of autophagy is a feasible approach (Desantis et al., 2018; Yang et al., 2020). Currently, various TCM compounds are known to exert anti-tumor effects through the targeted regulation of autophagy. Qianlie Xiaozheng decoction, a TCM formula, was shown to induce autophagy in human prostate cancer cells via inhibition of the Akt/mTOR Pathway (Xu et al., 2018). Furthermore, Wang et al. showed that BaBaoDan could inhibit tumor growth and increase the chemosensitivity of cisplatin in non-small-cell lung cancer *in vivo* and *in vitro* by facilitating autophagosome formation (Wang et al., 2019a). Here, several methods were used to explore the anticancer effects of SMBJD on MM *in vivo* and *in vitro*, in order to provide a scientific basis for the treatment of MM with SMBJD.

## Materials and Methods

### Preparation of SMBJD Water Extracts

SMBJD is a classic prescription composed of *Actaea heracleifolia (Kom.) J. Compton* (Shengma); *Marsdenia tenacissima* (Roxb.) *Moon* (Tongguanteng); *Carapax Trionycis* (Biejia); *Angelica sinensis* (Oliv.) *Diels* (Danggui); *Strobilanthes cusia* (Nees) *Kuntze* (Qinghdai); and *Glycyrrhiza uralensis Fisch. ex DC* (Gancao) combined in a ratio of 35:40:10:5:5:5 (Table 1). It was purchased from and processed by the Chinese pharmacy of the Affiliated Hospital of Nanjing University of Chinese Medicine. All of the materials (100 g) were soaked in 2 L of double-distilled water for 30 min. Then, the mixture was boiled for 1 h, and the liquid was filtered with gauze. The extraction procedure was repeated twice, and the extracted solutions were mixed. For the *in vivo* studies, the twice-extracted SMBJD solution was concentrated to an appropriate concentration for use. For the *in vitro* studies, it was concentrated to 100 ml; the resulting liquid concentration was 1.0 g/ml. After autoclaving and filtering through a 0.22 μm filter, the liquid was stored at −20°C.

**TABLE 1:** The ingredients of SMBJD (^*^Pharmacopoeia of People’s Republic of China, 2020 Edition).

Herb name	Place of origin	Part used	Traditional effect*	Dosage(g)
*Actaea heracleifolia* (kom.) *J. Compton* (Shengma)	Shanxi	Root, stem	Clearing heat, removing toxicity, promoting yang qi	35
*Marsdenia tenacissima* (Roxb.) *moon* (Tongguanteng)	Yunnan	Stem	Clearing heat, removing toxicity, relieivng cough and asthma, expectorant, promoting lactation	40
*Carapax trionycis* (*B*iejia)	Henan	Carapace	Nourishing yin, anti-febrile, softening hardness	10
*Angelica sinensis* (Oliv.) *diels* (Danggui)	Yunnan	Root	Supplementing blood, regulating mensturation, relieving pain, relieving constipation	5
*Strobilanthes cusia* (Nees) *kuntze* (Qingdai)	Fujian	Leaf, stem	Clearing heat, removing toxicity, cooling blood, purging fire, arresting convulsion	5
*Glycyrrhiza uralensis Fisch.ex* DC (Ganco)	Neimanggu	Root, stem	Tonifyin spleen, reinforcing qi, clearing heat, removing toxicity, relieving cough and asthma, expectant, relieving pain	5

### High-Performance Liquid Chromatography for SMBJD Quality Control

The five main ingredients in SMBJD (1 g/ml) were determined using an Agilent 1,260 liquid chromatography system. Briefly, 10 μL of SMBJD was injected into the apparatus with an auto sampler. Chromatographic separation was performed at a flow rate of 1 ml/min with an Agilent Zorbax SB-C18 column (4.6 × 250 mm, 5 μm). The mobile phase was composed of solvent A (0.1% phosphoric acid) and solvent B (acetonitrile). The linear gradient solutions were as follows: 2–18% solvent B for 0–8 min, 18% solvent B for 8–20 min, 18–25% solvent B for 20–30 min, 25–45% solvent B for 30–40 min, 45–60% solvent B for 40–50 min, and 60–2% solvent B for 50–51 min. The separation temperature was 40°C, with a detection wave length of 230 nm.

### Animal Xenograft Model and Treatments

Female BALB/c nude mice (aged 4–6 weeks, average weight 20 g) were purchased from the Animal Experiment Center of Zhejiang Province, China. The mice were bred in a specific pathogen-free environment and fed with sterile food and water. After seven days, six mice were randomly selected as the control group, and the remaining 34 mice were used as the experimental group. H929 cells in the logarithmic growth phase were collected and adjusted to a concentration of 5 × 10^7^ cells/ml. Each mouse in the experimental group was injected with 0.2 ml (1 × 10^7^ cells) into the left forelimb. On day 18 post-inoculation, the number of mice in which the tumor volume reached about 80–100 mm^3^ was counted, and they were divided into three groups randomly (six mice each group): the model group (double-distilled water, 0.1 ml/10 g via gavage, once per day and NS, 0.1 ml/10 g via tail vein injection, twice a week); the SMBJD group (SMBJD 60.67 mg/g, 0.1 ml/10 g via gavage, once per day and NS, 0.1 ml/10 g via tail vein injection, twice a week); the bortezomib group (double-distilled water, 0.1 ml/10 g via gavage, once per day and bortezomib, 0.50 μg/g, 0.1 ml/10 g via tail vein injection, twice a week). The treatment of the blank group was consistent with that of the model group. The length and width of tumors were measured every three days, and tumor volumes were calculated using the following formula: volume = (length × width^2^)/2. The mental states of mice were observed. After seven days of treatment, mice were anesthetized on the eighth day and blood was collected through the retro-orbital sinus, after which they were sacrificed by cervical dislocation. The tumors were weighed and measured. Mice were dissected to collect the liver, spleen, and kidneys. The tumor growth inhibition rate (%) was calculated as follows (1-weight of experiment group tumor/weight of model group tumor) × 100%. The experiments were approved by the Experimental Animal Ethics Committee of Nanjing University of Chinese Medicine.

### Hematoxylin and Eosin Staining

Mouse liver, spleen, and kidney were immediately fixed with 4% paraformaldehyde solution for 24 h and embedded in paraffin. The paraffin mass was sliced into 4 µm paraffin sections. After deparaffinization, sections were stained with Harris hematoxylin solution (Servicebio, Wuhan, China) for 3–8 min and then washed with distilled water, differentiated with 1% hydrochloric acid alcohol (Servicebio, Wuhan, China) for 20 s, and washed with distilled water again. Sections were then turned back to blue using 0.6% ammonia water (Servicebio, Wuhan, China) and washed with running water, before being stained with 0.5% eosin (Servicebio, Wuhan, China) for 1–3 min. Finally, the sections were dehydrated with an alcohol gradient and permeabilized with xylene (Servicebio, Wuhan, China); the slides were sealed and then scanned using a Pannoramic 250 Flash III (3dhistech, Budapest, Hungary).

### Immunohistochemistry Staining

Tumor tissues from mice were immediately fixed with 4% paraformaldehyde solution for 24 h and embedded in paraffin. The paraffin mass was sliced into 4 μm paraffin sections and dewaxed, then antigen retrieval was performed with 10 mM of boiled citrate buffer (pH 6.0) (Servicebio, Wuhan, China) for 15 min in a microwave oven. This was followed by washing three times in phosphate-buffered saline (PBS) after cooling naturally, for 5 min each time. Then, sections were incubated with 3% hydrogen peroxidase in the dark at room temperature for 25 min to block endogenous peroxidase, and washed three times in PBS for 5 min each time. Sections were then blocked with 3% bovine serum albumin for 30 min and incubated with rabbit monoclonal primary antibodies against P62 (1:20–1:200, Proteintech, IL, United States), LC3 (1:50–500, Proteintech, IL, United States), cleaved caspase-3 (1:200, CST, MA, United States), ERK1/2 (1:400–1,600, CST, MA, United States), phosphorylated ERK1/2 (1:400, CST, MA, United States), Ser2448 mTOR (1:100, CST, MA, United States), and phosphorylated Ser2448 mTOR (1:100, CST, MA, United States) overnight at 4°C. After washing three times in PBS again, for 5 min each time, they were incubated with the appropriate secondary antibodies (Servicebio, Wuhan, China) for 50 min and then washed again. DAB chromogenic solution (Servicebio, Wuhan, China) was used for color development. Harris hematoxylin was used to stain nuclei. Finally, sections were dehydrated with an alcohol gradient and permeabilized with xylene, then scanned with a Pannoramic 250 Flash III.

### Cells and Cell Culture

Human MM cell lines H929 and U266 were supplied by the Central Laboratory of the Affiliated Hospital of Nanjing University of Chinese Medicine. Both H929 and U266 cells were cultured in Roswell Park Memorial Institute 1,640 (RPMI-1640) (Gibco, CA, United States) medium containing 10% fetal bovine serum (FBS) (VivaCell, Shanghai, China) in a humidified incubator with 5% CO_2_ at 37°C.

### Cell Viability

A colorimetric MTT assay was performed to evaluate the growth inhibitory effect of the indicated SMBJD treatments. Briefly, H929 and U266 cells (1 × 10^4^/well) were seeded in 96-well plates (Jet Biofil, Guangzhou, China) with 90 μL of their respective media, followed by the addition of 10 μL of SMBJD at a concentration of 1, 2, 4, 8, and 16 mg/ml for 36 or 48 h. The marginal pores were filled with 150 μL of PBS (Gibco, CA, United States). Thereafter, 20 μL of MTT (5 mg/ml) (Sigma, MO, United States) was added to each well during the last 4 h of incubation. The cells were centrifuged at 1,000 rpm for 3 min and the supernatant was removed. Subsequently, 150 μL of dimethyl sulfoxide (Gibco, CA, United States) was added to each well. The plate was vibrated for 10 min. Absorbance was measured at 490 nm using a microplate reader (BioTek Instruments, VT, United States). At least three independent experiments were performed, and each sample was assessed five times using the following formula: cell viability = absorbance of test sample/absorbance of control sample × 100%.

### Ad-GFP-LC3 Transfection

H929 and U266 cells were seeded in a six-well plate at a density of 1 × 10^4^/ml, infected with 2 µL of recombinant adenovirus expressing GFP-LC3 (Ad-GFP-LC3) (Sciben, Nanjing, China) for 24 h, and then treated with different doses of SMBJD for 36 h. After that, cells were centrifuged and washed twice with PBS, and 10–15 µL of each cell suspension was taken as a sample. An upright phase-contrast fluorescence microscope (Nikon80i, Nikon, Tokyo, Japan) was used to select an excitation wavelength of 380 nm to observe the autophagy state of the cells. The numbers of green fluorescent spots were taken as indicators of the presence of autophagosomes.

### Flow Cytometry

H929 and U266 cells with corresponding treatments in each group were stained with annexin V-FITC/propidium iodide (PI). After 36 h of treatment with different doses of SMBJD, rapamycin (1 nmol/L), and SMBJD + rapamycin in 6-well plates (Jet Biofil, Guangzhou, China) with a density of 1 × 10^4^/ml, cells were centrifuged at 1,000 rpm for 3 min and the supernatant was discarded. Cells were washed with PBS twice, and annexin V-FITC/PI staining was conducted according to the protocol of the apoptosis kit (MULTI SCIENCES, Nanjing, China). Cells were then re-suspended in 500 μL of 1 × binding buffer (MULTI SCIENCES, Nanjing, China), followed by incubation with 10 μL of annexin V-FITC and 10 μL of PI for 15 min at 4°C in a dark environment. Finally, the cells’ apoptosis rates were detected by flow cytometry (BD, NJ, United States).

### Western Blot

Tissue proteins were extracted from mouse tumors and ground after the addition of RIPA lysis buffer, followed by centrifuging at 20 g for 20 min at 4°C. The supernatant was collected for determination of its concentration.

For the analysis of cell proteins, the cells were seeded in a six-well culture plate at a density of 1 × 10^5^/ml. SMBJD, 3-MA (0.25 mmol/L), Baf-A1 (0.5 nmol/L), rapamycin (1 nmol/L), SMBJD + Baf-A1, and SMBJD + rapamycin were added at the specified doses, and the samples were incubated for 36 h. Cells were then centrifuged, washed, harvested, and lysed using RIPA lysis buffer containing a protease inhibitor cocktail for 15 min. The lysates were ultrasonicated for 60 s (four times for 15 s each) and centrifuged at 20 g for 20 min at 4°C, and the supernatants were collected for concentration determination.

Finally, the concentrations of the tissue and cell proteins were determined. Equal amounts of protein (30 μg) from each sample were separated by sodium dodecyl sulfate polyacrylamide gel electrophoresis and transferred to polyvinylidene fluoride membranes. The membranes were blocked with 5% fat-free milk with 0.1% Tween-20 in 0.02 ml of TBS buffer (TBST) for 1 h and incubated with primary antibodies against P62 (1:1,000–4,000, Proteintech, IL, United States), LC3 (1:600–2,500, Proteintech, IL, United States), Bax (1:1,000, CST, MA, United States), Bcl-2 (1:1,000, CST, MA, United States), caspase-3 (1:1,000, CST, MA, United States), cleaved caspase-3 (1:1,000, CST, MA, United States), ERK1/2 (1:10,000, Abcam, Cambridge, United Kingdom), phosphorylated ERK1/2 (1:5,000–10,000, Abcam, Cambridge, United Kingdom), Ser2448 mTOR (1:500–2,000, Sciben, Nanjing, China), and phosphorylated Ser2448 mTOR (1:500–2,000, Sciben, Nanjing, China) at 4°C overnight. After incubation with primary antibodies, the membranes were washed with TBST four times and incubated with the appropriate horseradish peroxidase-conjugated secondary antibody for 1 h. Membranes were then washed and examined by ChemiDOC XRS+ (BIO-RAD, CA, United States). Equal protein loading was confirmed by probing blots with the anti-*β*-actin antibody. Optical density values were measured using Image Lab.

### Statistical Analysis

Statistical analyses were conducted with SPSS 22.0. Measurement data were calculated as means and standard deviations. Differences between groups were analyzed using *t*-test or one-way analysis of variance. Numerical data were statistically analyzed by χ^2^-test. A *p*-value lower than 0.05 (*p* < 0.05) was considered as evidence of statistical significance.

## Results

### HPLC Chromatograms of SMBJD

**FIGURE 1:**
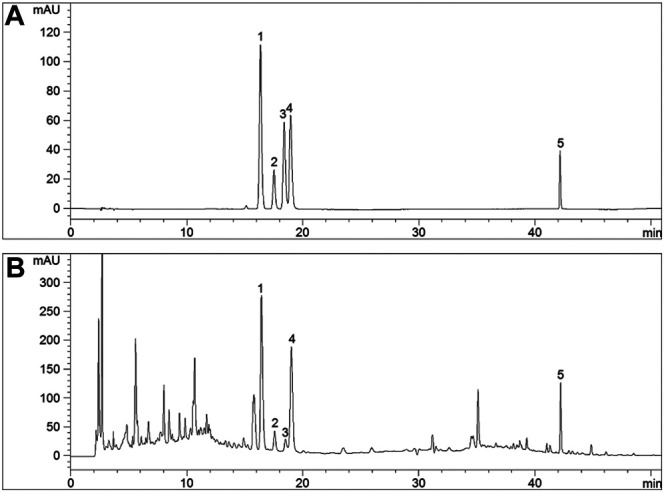
The quality control of SMBJD evaluated by HPLC. **(A)** Representative HPLC chromatograms of references. **(B)** Representative HPLC chromatograms of SMBJD. (1. liquiritin; 2. ferulic acid; 3. cimifugin; 4. isoferulic acid; 5. glycyrrhizic acid).

The five major components of SMBJD (1.0 g/ml) were analyzed by HPLC (Figure 1B). The liquiritin, ferulic acid, cimifugin, isoferulic acid, and glycyrrhizic acid contents were 1,102.20, 229.24, 67.14, 1,534.73, and 1,560.82 μg/g, respectively.

### SMBJD Exerts Anti-Tumor Activity in H929 Xenograft Model and Promotes Hemogram Recovery

**FIGURE 2:**
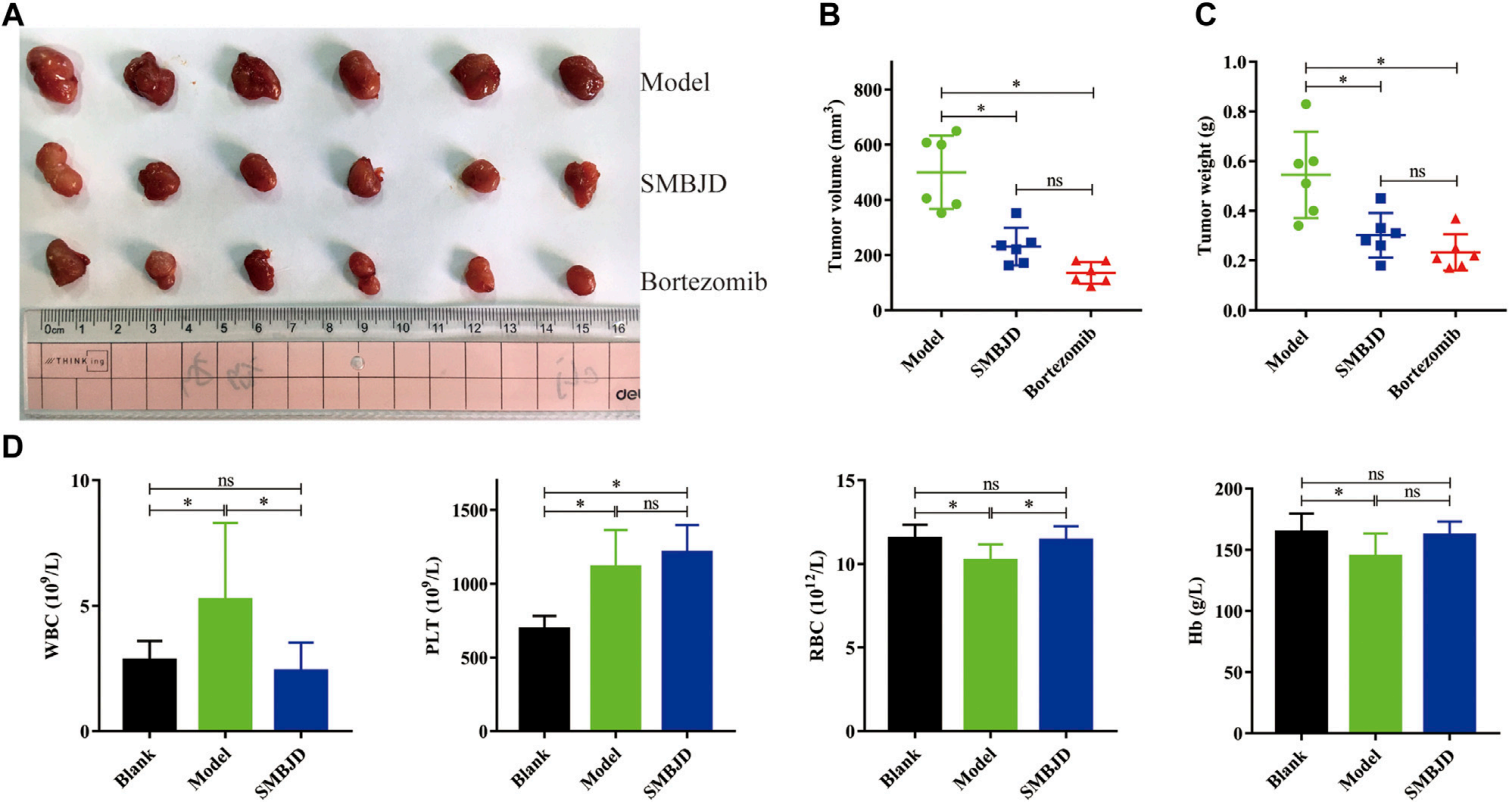
SMBJD exerts anti-tumor activity in H929 xenograft model, promotes the recovery of hemogram. BALB/c nude mice were subcutaneously implanted with 1 × 10^7^ H929 cells and divided into three groups randomly (model group, SMBJD group, and bortezomib group) after 18 days. **(A)** Representative image of tumors from mice in each group. **(B)** The final tumor volume were recorded and calculated. **(C)** The final tumor weight of each mouse was weighed after harvest. **(D)** After seven days treatment of SMBJD, the blood routine of each mouse was tested. Data are expressed as the means ± SD (^ns^ *P* > 0.05, *p < 0.05 compared with model group).

To further investigate the effects of SMBJD on tumor growth, a mouse model with a H929 xenograft was constructed. After seven days of continuous treatment with SMBJD and bortezomib, respectively, the results showed that SMBJD and bortezomib inhibited tumor volume growth (Figure 2A). However, there was no difference in tumor volume and tumor weight between the SMBJD group and the bortezomib group (Figures 2B,C). The tumor growth inhibition rates in the SMBJD group and the bortezomib group were 44.65 and 57.19%, respectively. Furthermore, routine blood tests showed that WBC and PLT were higher in the model group than in the blank group, whereas RBC and Hb were lower compared with the blank group. In the SMBJD group, RBC was higher and WBC was lower compared with the model group. Notably, there were no statistical differences in WBC, RBC, and Hb between the SMBJD group and the blank group; differences were observed only for PLT (Figure 2D).

### SMBJD is Non-Toxic

**FIGURE 3:**
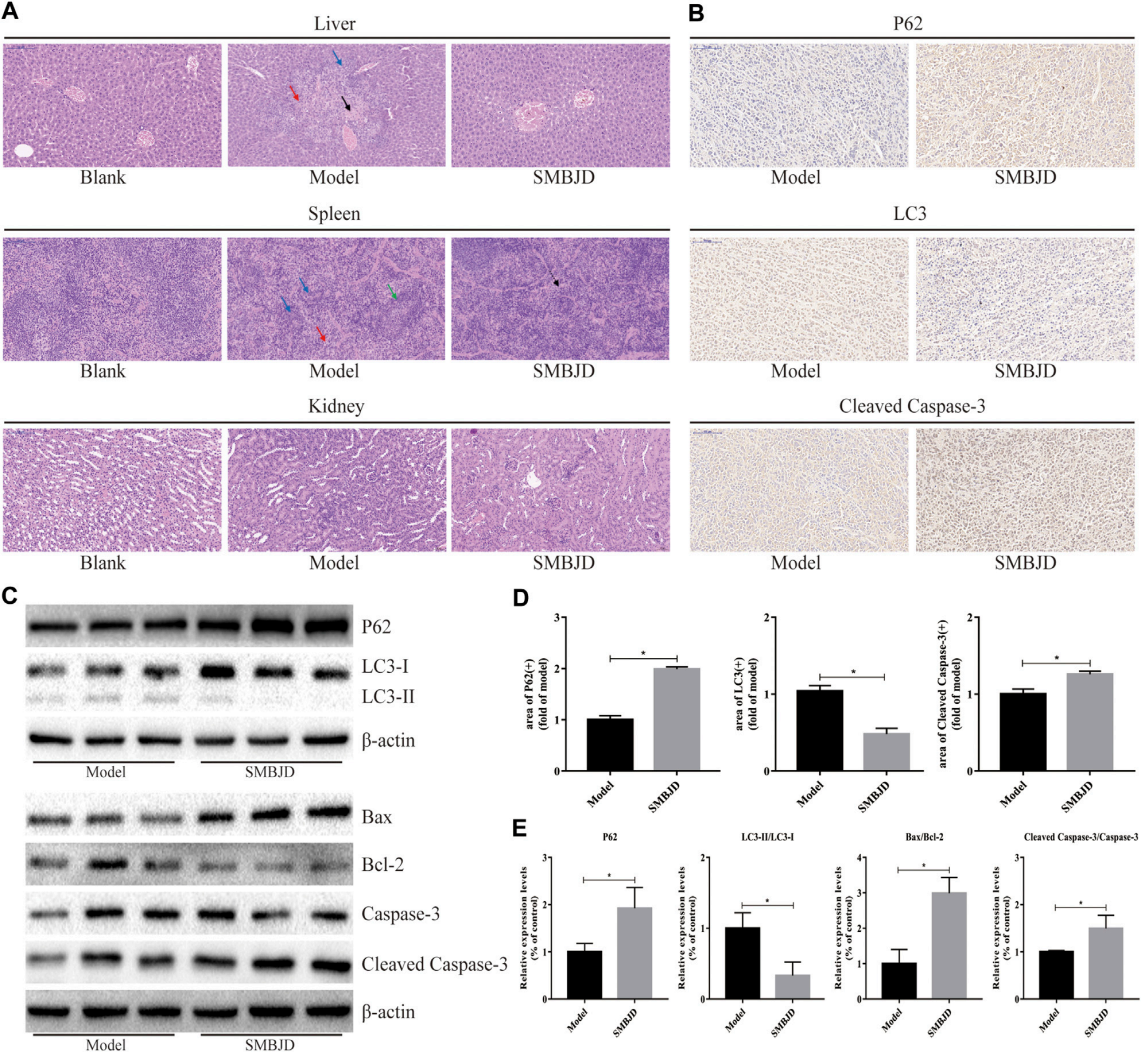
SMBJD is safe and could inhibit the expression of autophagy-related proteins, promote apoptosis-related proteins *in vivo*. **(A)** Representative pictures of peer group mouse liver, spleen, and kidney after HE staining (× 200), scale bar = 100 µm. **(B)** The expression levels of P62, LC3, and cleaved caspase-3 in transplanted tumors were detected using immunohistochemistry (× 200), scale bar = 100 µm. **(C)** Extracts from transplanted tumors were analyzed for autophagy-related and apoptosis-related proteins expression by western blot. **(D)** The corresponding expression of P62, LC3, and cleaved caspase-3 levels in tumor tissue are shown as histograms. **(E)** The corresponding autophagy-related and apoptosis-related proteins expression by western blot are shown as histograms. (*p < 0.05 compared with the model group).

In order to examine the effects of SMBJD on organ function, HE staining of the liver, spleen, and kidneys of mice was performed. The results showed significant liver damage in the model group. Hepatocellular necrosis of different extents (black arrow, liver, Figure 3A) was observed, accompanied by a small amount of fibroblast hyperplasia repair (red arrow, liver, and Figure 3A), and some neutrophil cells infiltration at the edge of the necrotic focus (blue arrow, liver, and Figure 3A). Importantly, the liver pathology of the blank group and the SMBJD group showed no abnormalities.

Regarding splenic pathological changes, there was no obvious pathological damage in the blank group. In the model group, white pulp indicated obvious atrophy (green arrow, spleen, and Figure 3A). Moreover, red medullary trabecular structure and tissue cells proliferation were also observed (red arrow, spleen, and Figure 3A). In the SMBJD group, only slight red medullary trabecular structure hyperplasia and tissue cell proliferation lymphocytosis were observed (black arrow, spleen, and Figure 3A).

Finally, the pathological changes in the kidneys of the mice in each groups were examined, while there was no obvious pathological change in kidney structures in each group.

The above results indicate that SMBJD can reduce organ damage in mice, that is, these doses of SMBJD used here were non-toxic from this perspective.

### SMBJD Inhibits the Expression of Autophagy-Related Proteins and Promotes Apoptosis-Related Proteins *in vivo*

Immunohistochemistry and western blotting were used to detect the expression levels of autophagy- and apoptosis-related proteins in tumor tissues, for the purpose of verifying the mechanism of SMBJD’s anti-tumor effects *in vivo*. The immunohistochemical analysis was used to detect P62, LC3, and cleaved caspase-3. The results showed that SMBJD decreased positive LC3 expression and increased positive P62 and cleaved caspase-3 expression (Figure 3B). Furthermore, western blot results showed that SMBJD inhibited the process of autophagy, as evidenced by decreased levels of LC3-II/LC3-I and increased levels of P62, whereas the upregulation of Bax/Bcl-2 and cleaved caspase-3/caspase-3 indicated that SMBJD promoted apoptosis simultaneously (Figure 3C). These results indicate that SMBJD probably inhibited autophagy and induced apoptosis to exert its anti-tumor function.

### SMBJD Represses the Growth of H929 and U266 Cells

**FIGURE 4:**
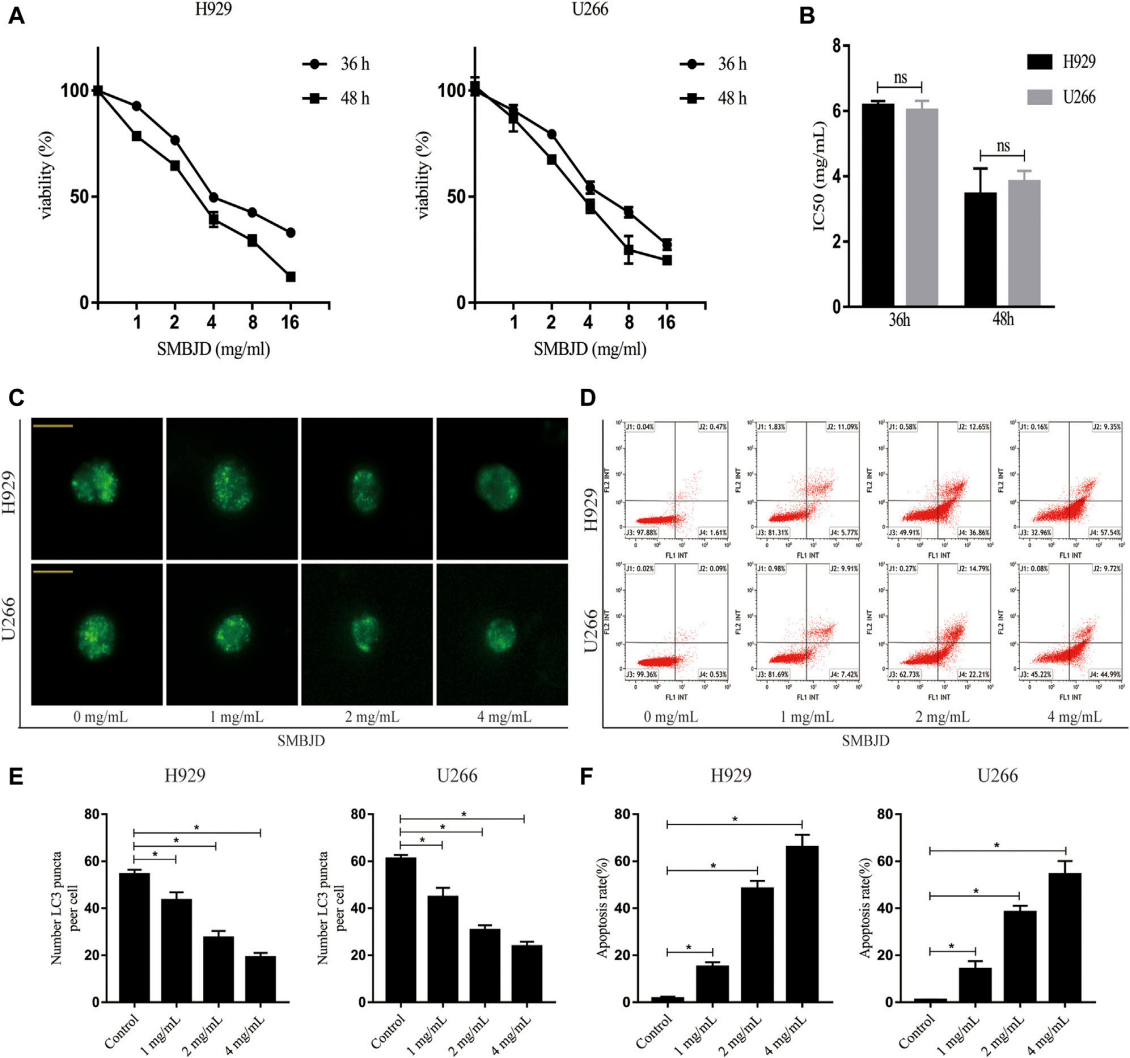
SMBJD represses the growth, inhibits autophagy, and promotes apoptosis of H929 and U266 cells. **(A)** The cell viability of H929 and U266 cells after different concentrations and time of SMBJD treatment were analyzed with MTT assay. **(B)** IC50 value of H929 and U266 cells after SMBJD intervention. **(C)** H929 and U266 cells were transiently transfected with Ad-GFP-LC3 for 24 h. Then the cells were cultured with SMBJD for another 36 h. The formation of GFP-LC3 puncta were examined by a confocal microscope and typical images were presented (× 400), scale bar = 2 µm. **(D)** Annexin V/PI staining of H929 and U266 cells apoptosis rate treated with SMBJD for 36 h **(E, F)** The quality graphs of C and D. Data are expressed as the means ± SD (^ns^ *P* > 0.05 compared with each other, *p < 0.05 compared with control group).

MM cell lines H929 and U266 were pretreated with various concentrations of SMBJD (0–16 mg/ml) for 36 and 48 h. The MTT assay showed that SMBJD inhibited the proliferation of both H929 and U266 cells in a time and dose-dependent manner (Figure 4A). The median inhibitory concentration (IC_50_) values for the H929 cells were 6.16 ± 0.14 mg/ml for 36 h and 3.44 ± 0.79 mg/ml for 48 h. For the U266 cells, the IC_50_ values were 6.02 ± 0.29 mg/ml and 3.83 ± 0.33 mg/ml for 36 and 48 h, respectively. Thus, there were no clear differences in IC_50_ values between H929 and U266 cells regardless of 36 or 48 h SMBJD treatment (Figure 4B). These data indicate that SMBJD has cytotoxic effects.

### SMBJD Inhibits Autophagy and Promotes Apoptosis of MM Cell Lines H929 and U266

**FIGURE 5:**
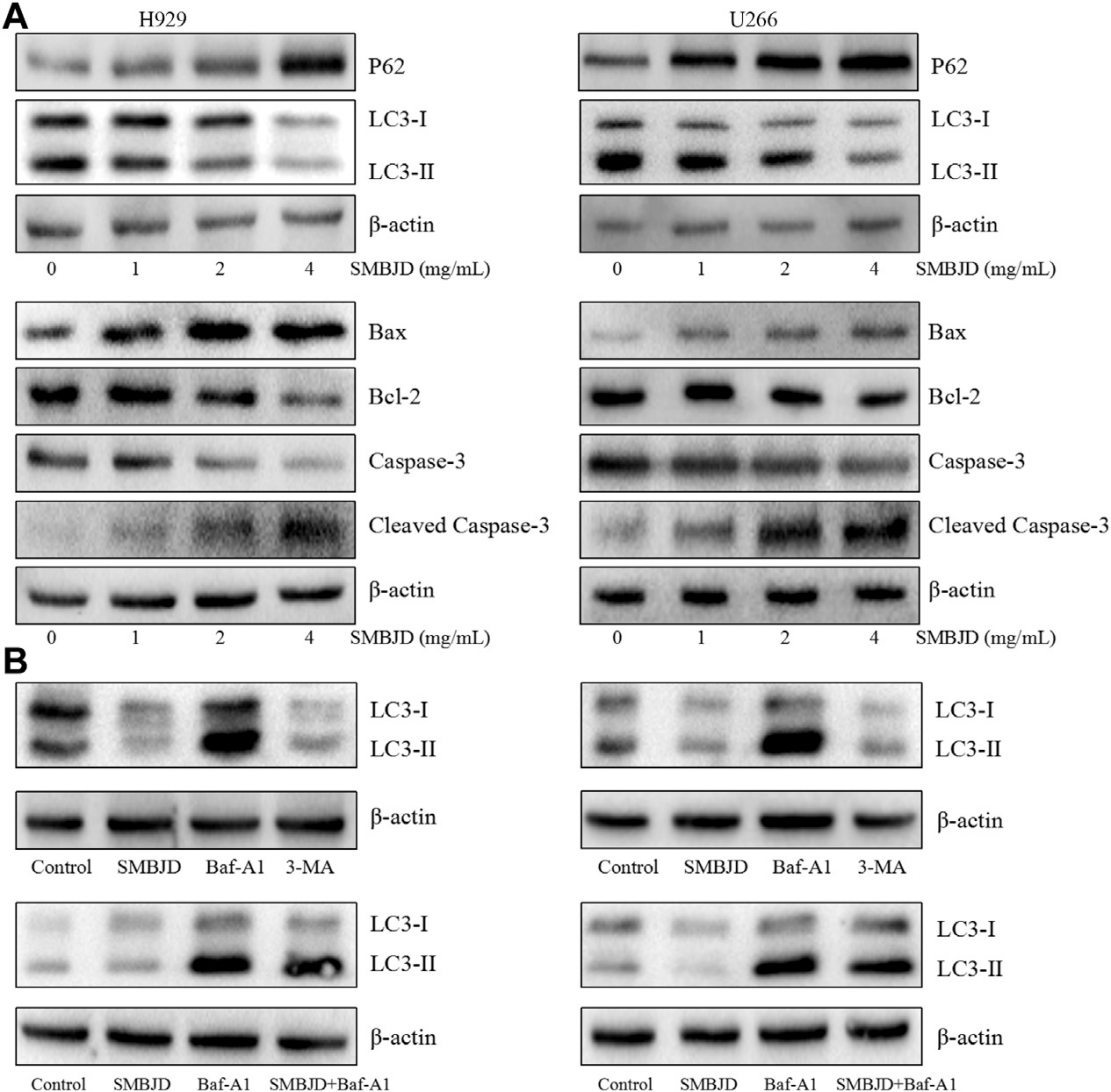
SMBJD inhibits autophagy-related proteins and promotes apoptosis-related proteins of multiple myeloma cell lines H929 and U266 **(A)** Western blot analysis of autophagy-related and apoptosis-related proteins expression after SMBJD administration for 36 h in both cells. **(B)** Western blot analysis of LC3 expression after SMBJD, 3-MA, Baf-A1, and SMBJD + Baf-A1 administration for 36 h in H929 and U266 cells.

To further determine the reason for cell death, fluorescence-labeled autophagosomes were counted after infection with Ad-GFP-LC3 to detect levels of autophagy. There was a substantial accumulation of autophagosomes and autophagolysosomes in both H929 and U266 cells. However, numbers of GFP-LC3 puncta were decreased after treatment with SMBJD (Figure 4C). Western blot analysis showed that the expression levels of LC3-II/LC3-I proteins decreased whereas those of P62 proteins increased (Figure 5A). However, as autophagy is a dynamic process, the reduction in LC3-II/LC3-I could indicate that SMBJD inhibited the formation of autophagosomes or that it promoted the degradation of autophagolysosomes. To clarify this point, 3-MA, an early–stage autophagic inhibitor and Baf-A1, a late-stage autophagic inhibitor that inhibits the combination of autophagosomes and lysosomes, were used to test the expression of LC3-II/LC3-I. SMBJD and 3-MA treatment resulted in a decrease in LC3-II/LC3-I compared with the control group, whereas Baf-A1 contributed to the upregulation of LC3-II/LC3-I. Moreover, SMBJD could not reverse the augmentation of LC3-II/LC3-I produced by Baf-A1 in the SMBJD + Baf-A1 group (Figure 5B).

In addition, flow cytometry was used to determine whether SMBJD affected apoptosis in H929 and U266 cells. The results showed that SMBJD increased the rate of apoptosis in a concentration-dependent manner (Figure 4D). Western blotting was used to analyze the expression of apoptosis-related proteins. The results showed that cleaved caspase-3/caspase-3 and pro-apoptotic Bax/anti-apoptotic Bcl-2 were upregulated (Figure 5A). Collectively, these results verify that SMBJD inhibits autophagy but promotes apoptosis in H929 and U266 cells.

### SMBJD Induces Apoptosis Mediated by Inhibition of Protective Autophagy

**FIGURE 6:**
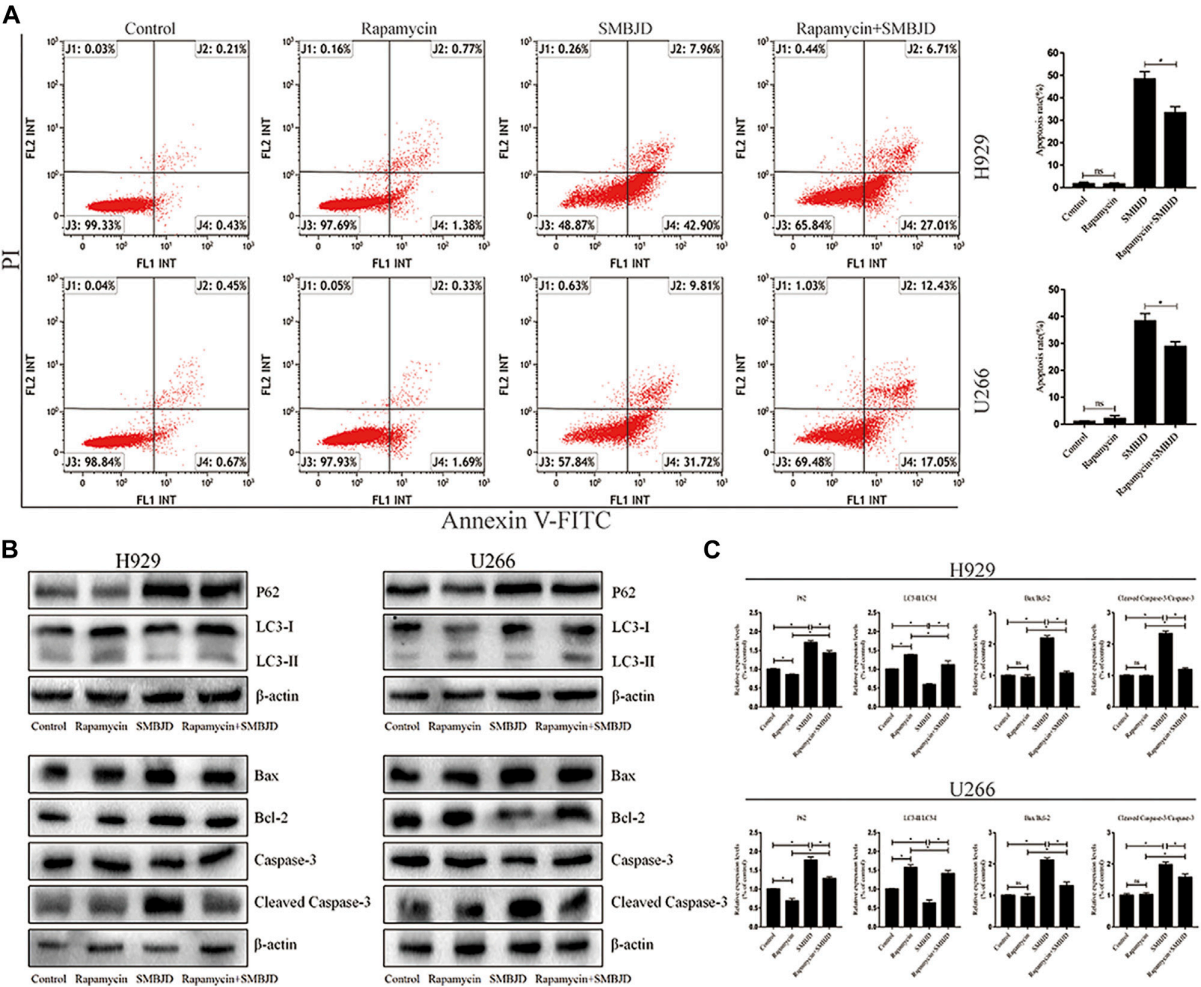
SMBJD induces apoptosis mediated by protective autophagy. **(A)** Apoptosis rates in H929 and U266 cells were assayed by flow cytometry under SMBJD, rapamycin and SMBJD + ramamycin intervention. **(B)** Western blot analysis of autophagy-related and apoptosis-related proteins expression in each group. **(C)** The quality graphs of B. Data are expressed as the means ± SD ( ^ns^ *P* > 0.05, *p < 0.05 compared with each other).

To further validate whether the apoptosis induced by SMBJD was mediated by autophagy, rapamycin, an autophagy agonist, was applied to H929 and U266 cells at a concentration (1 nmol/L) that has no effect on apoptosis. First, flow cytometry was used to analyze apoptosis in H929 and U266 cells after rapamycin treatment. In H929 and U266 cells, apoptosis declined after treatment with rapamycin + SMBJD compared with SMBJD alone (Figure 6A). Western blotting was used to examine the expression of related proteins. The results showed that rapamycin downregulated P62 expression and upregulated the expression of LC3-II/LC3-I (Figure 6B). Furthermore, there was no obvious difference in the expression of apoptosis-related proteins in the rapamycin group compared with the control group, whereas the expression levels of Bax/Bcl-2 and cleaved caspase-3/caspase-3 decreased in the rapamycin + SMBJD group compared with the SMBJD group (Figure 6B). These results suggest that apoptosis induced by SMBJD in H929 and U266 cells could be decreased by rapamycin, thereby proving that the promotion of apoptosis by SMBJD is mediated by autophagy.

### SMBJD Induces Autophagy-Mediated Apoptosis via Upregulation of ERK/mTOR Signaling Pathway *in vivo* and *in vitro*

**FIGURE 7:**
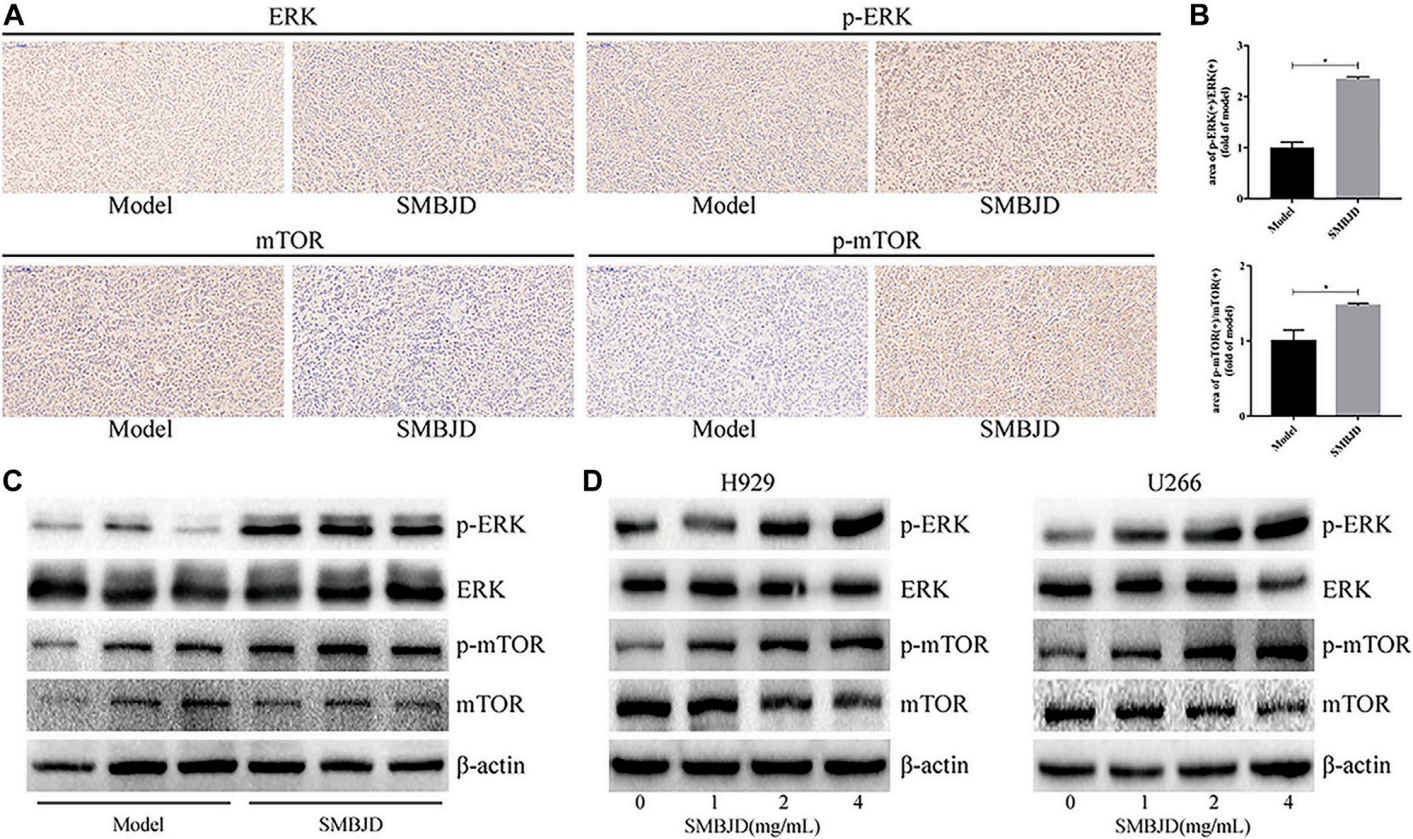
Erk/mTOR pathway participated in regulating autophagy and apoptosis in H929 and U266 cells after SMBJD intervention. **(A)** The expression levels of p-Erk, Erk, p-mTOR, and mTOR in transplanted tumors were detected by immunohistochemistry (× 200), scale bar = 100 µm. **(B)** The quality graphs of A. **(C, D)** The expression levels of related proteins in tumor tissue and cells tested by western blot.

In order to further elucidate the mechanism of SMBJD, several signaling pathway molecules were assayed using immunohistochemistry and western blotting. Based on levels of activated phosphorylated ERK and phosphorylated Ser2448 mTOR, SMBJD exposure increased ERK and Ser2448 mTOR activation both *in vivo* and *in vitro* (Figures 7A–D). Hence, these data indicate that SMBJD induced autophagy-mediated apoptosis via activation of the ERK/mTOR signaling pathway.

## Discussion

SMBJD has been widely used for clinical treatment for more than 1800 years. Based on the basic theories of TCM and Chinese medicine pharmacology, it was used as an adjuvant treatment for hematological malignancies and showed significant clinical efficacy. This study demonstrates that SMBJD inhibits protective autophagy and induces autophagy-mediated apoptosis to exert an anticancer effect through the ERK/mTOR pathway.

In the present study, SMBJD (60.67 mg/g) and bortezomib (0.5 μg/g) were used to treat a H929 xenograft mouse model for seven days. The results showed that SMBJD and bortezomib could inhibit tumor growth. Likely due to the short administration time of bortezomib, there was no statistical difference in tumor volume and tumor weight between the SMBJD group and the bortezomib group. Patients with MM often have multiple osteolytic damage, hypercalcemia, anemia, kidney damage, and infection, owing to the inhibition of immunoglobulin production (Rajkumar, 2016; Rajkumar, 2020). Not surprisingly, there were abnormal hemograms in the model group, with higher WBC and PLT, and lower RBC and Hb compared with the blank group. This led to speculation that transplantation of human H929 cells into mice would cause inflammation and bone marrow suppression. After treatment with SMBJD, there were no significant differences in WBC, RBC, or Hb in the SMBJD group compared with the blank group, which indicated that SMBJD could promote hemogram recovery and improve the growth status of mice. The toxicity of SMBJD was also considered. Chinese medicines have side effects when the prescribed dosage is exceeded, including organ damage. To clarify this point, HE staining analysis of the liver, spleen, and kidneys of mice from each group was performed. The results showed that SMBJD could reduce the liver, spleen, and kidney damage caused by transplantation of H929 cells.

Next, in order to verify whether SMBJD exerts its anticancer effect via modulation of autophagy, the expression of LC3-II/LC3-I, which indicates the level of autophagy, was tested in tumor tissues; it was downregulated after SMBJD treatment (Wang et al., 2008; Runwal et al., 2019), whereas P62, which forms a complex with LC3-II and is then degraded in the lysosome in the late stage of autophagy, increased after SMBJD treatment (Bjorkoy et al., 2005). Accumulation of P62 results in either a reduction in autophagy activity or a block in the combination of autophagosome and lysosome (Li et al., 2020). Thus, these results indicate that SMBJD can inhibit the autophagy process. However, there is another programmed cell death pathway, apoptosis. There are highly interconnected signaling networks between autophagy and apoptosis, although they often exhibit contradictory roles in tumor cells growth (Maiuri et al., 2007; Booth et al., 2014; Marino et al., 2014). Analysis of the expression of apoptosis-related proteins showed an obvious increase in the ratio of Bax/Bcl-2 and cleaved caspase-3/caspase-3 in the SMBJD treatment group. All the animal data suggest that SMBJD exerts its anticancer effect by inhibiting autophagy and promoting apoptosis, probably at the same time.

To further verify the results of the *in vivo* experiments, H929 and U266 cell lines were selected for *in vitro* experiments. There was a loss of viability in H929 and U266 cells after treatment with SMBJD (0–16 mg/ml) for 36 and 48 h. Moreover, there were no statistical differences in IC_50_ values for H929 and U266 cells treated for 36 or 48 h. It has been verified that SMBJD could affect the processes of autophagy and apoptosis, thereby regulating its anti-tumor effects in *in vivo* experiments. The numbers of GFP-LC3 puncta and the flow cytometry results after SMBJD treatment corroborated this finding. The western blot results also demonstrated that SMBJD could repress autophagy and aggravate apoptosis by regulating the expression of P62, LC3, Bax, Bcl-2, and cleaved caspase-3 in H929 and U266 cells. However, the expression of P62 and LC3 represents a dynamic process in the occurrence of autophagy (Glick et al., 2010; Parzych and Klionsky, 2014). To further distinguish the effects of SMBJD on autophagy, H929 and U266 cells were pre-exposed to 3-MA and Baf-A1 separately. SMBJD and 3-MA both led to a decrease in LC3-II/LC3-I, whereas there was an accumulation of LC3-II in the presence of Baf-A1. However, co-incubation with SMBJD and Baf-A1 did not result in any difference in levels of LC3-II/LC3-I compared with Baf-A1 alone. These data indicate that SMBJD inhibits autophagosome formation and does not activate autophagic flux.

Autophagy and apoptosis are the two main types of programmed cell death (Fuchs and Steller, 2015). The balance between autophagy and apoptosis is vital to the survival of myeloma cells. Under conditions of nutrient depletion, autophagy can protect MM cells from apoptosis (Zeng et al., 2012). Some drugs or the knockout of certain genes have been shown to inhibit autophagy so as to promote apoptosis-related myeloma cell death (Jarauta et al., 2016). Li et al. found that NUPR1 was highly expressed in MM patients, then, through silencing NUPR1, they observed the inhibition of autophagy and autophagy-mediated apoptosis in U266 and RPMI 8226 cells. Previous experiments in this study have shown that SMBJD could regulate the autophagy and apoptosis of U266 and H929 cells. For further research, rapamycin, an autophagy agonist, was used to intervene in H929 and U266 cells, alone and in combination with SMBJD. Considering the apoptosis-inducing effect of rapamycin itself on H929 and U266 cells, a concentration was chosen at which rapamycin could activate autophagy of the H929 and U266 cells with no effect on apoptosis. A reduced apoptosis rate was observed in the rapamycin + SMBJD group compared with the SMBJD group. It was not surprising that the western blot assay showed a lower ratio of Bax/Bcl-2 and cleaved caspase-3/caspase-3 in the rapamycin + SMBJD group compared with the SMBJD group. So far, the results have clarified that SMBJD-induced apoptosis is mediated by the inhibition of autophagy, which has a protective function in H929 and U266 cells.

It is well known that several signaling pathways are involved in the process of autophagy. For instance, mitophagy is mediated either by the Pink1–Parkin signaling pathway or the mitophagic receptors Nix and Bnip3, while the established master regulator of macroautophagy is mTOR (Ding and Yin, 2012; Feng et al., 2013; Dossou and Basu, 2019), a key protein regulating cell growth and proliferation (Hay and Sonenberg, 2004). One of its complexes, mTORC1, can accept multiple autophagy signals, thereby inhibiting cell autophagy (Noda, 2017). In this study, through using rapamycin, an inhibitor of mTOR, the inhibition of autophagy induced by SMBJD was reversed in the SMBJD + rapamycin group. This demonstrated that the molecular mechanism of SMBJD involved the activation of mTOR in H929 and U266 cells. Thereafter, several signaling molecules upstream of mTOR were detected through western blotting. The expression levels of phosphorylated ERK were increased after different treatment concentrations. The same trend was observed in tumor tissues. These data support a role for the ERK/mTOR in autophagy-mediated apoptosis. Taken together, the results of this study indicate that SMBJD represses autophagy and augments autophagy-mediated apoptosis through upregulation of the ERK/mTOR pathway *in vivo* and *in vitro*.

## Conclusion

This study is the first to demonstrate the molecular mechanisms of SMBJD in the treatment of MM both *in vivo* and *in vitro*. SMBJD could accelerate apoptotic death by inhibiting the protective autophagy of MM cells through the ERK/mTOR signaling pathway. These results provide a scientific basis for the clinical adjuvant use of SMBJD.

## Data Availability

The raw data supporting the conclusions of this article will be made available by the authors, without undue reservation.
